# DNA polymerase λ autoinhibition is relieved via Ku interaction during non-homologous end joining

**DOI:** 10.1093/nar/gkag114

**Published:** 2026-02-17

**Authors:** Brandon C Case, Leonardo Scoccia, Zhihan Zhao, Joseph J Loparo

**Affiliations:** Department of Biological Chemistry and Molecular Pharmacology, Blavatnik Institute, Harvard Medical School, Boston, MA 02115, United States; Department of Biology and Biotechnology, University of Pavia, Pavia 27100, Italy; Institute of Molecular Genetics–National Research Centre (IGM-CNR), Pavia27100, Italy; Department of Chemistry, Vanderbilt University, Nashville, TN 37235, United States; Department of Biological Chemistry and Molecular Pharmacology, Blavatnik Institute, Harvard Medical School, Boston, MA 02115, United States

## Abstract

DNA ends are frequently damaged during the formation of DNA double-strand breaks (DSBs). These ends must be repaired to enable ligation during non-homologous end joining (NHEJ). NHEJ uses several end processing factors to repair DNA ends within the short-range synaptic complex (SRC), including Polymerase λ (Pol λ) which performs gap fill-in. Pol λ possesses a Ku Binding Motif (KBM) within its BRCT domain that interacts with Ku and recruits it to the SRC. Here, we show that in addition to its role in recruitment, Ku also stimulates Pol λ polymerase activity at DSBs. Using a structural prediction approach and biochemical assays, we identify and characterize an autoinhibitory intramolecular interaction between the N-terminal BRCT and C-terminal catalytic domains of Pol λ. Furthermore, single-molecule approaches reveal that Ku increases both the binding rate of Pol λ to primer-template DNA and the rate of nucleotide incorporation, demonstrating that Ku releases Pol λ autoinhibition and stimulates its polymerase activity within the SRC during NHEJ. Combined, these data highlight how intricate protein–protein interactions within the SRC complex are critical to regulate end-processing and maximize the fidelity of DSB repair.

## Graphical Abstract

**Graphical Abstract:**
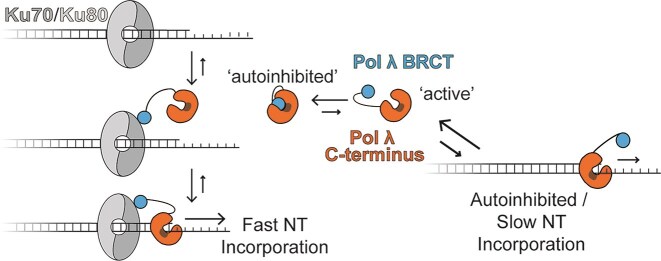

## Introduction

DNA double-strand breaks (DSBs) are extremely toxic lesions that threaten genome stability. Successful repair of DSBs is critical to ensure cell survival and faithful preservation of genetic information with defects in repair pathways leading to tumorigenesis and cell death [1]. Homologous recombination and non-homologous end joining (NHEJ) are the main pathways employed by cells to repair DSBs, with NHEJ resolving a vast majority of DSBs throughout the cell cycle in a template independent manner [2–4].

During NHEJ, broken DNA ends are recognized and aligned for ligation within the synaptic complex. Recognition of broken ends is initiated by the ring-shaped Ku70/Ku80 heterodimer (Ku), which rapidly binds DNA ends with high affinity [5]. DNA bound Ku functions as a recruitment hub for downstream NHEJ factors, interacting with different binding partners via their ‘Ku Binding Motifs’ (KBMs), which have been extensively characterized in a variety of crystal structures and biochemical work [6–10]. Subsequent to Ku binding, the DNA dependent protein kinase catalytic subunit (DNA-PKcs) is recruited to the DSB to protect DNA ends and phosphorylate several NHEJ core factors, including itself [11–16]. The holoenzyme DNA-PK, composed of Ku and DNA-PKcs, forms a homodimer tethering the two broken DNA ends together within the long-range synaptic complex (LRC) [17]. Once the LRC is formed, recruitment of XRCC4/DNA Ligase 4 (Lig4) and XRCC4-like factor (XLF) transitions the LRC to the short-range synaptic complex (SRC). Within the SRC, NHEJ factors span the DSB to enable ligation by bringing the ends within range of Lig4 [4, 17–19].

During DSB formation, DNA ends are often damaged which prevents their immediate re-ligation by Lig4 [20]. To repair these damages, various enzymes, including kinases, polymerases, and nucleases, are recruited to the SRC to prepare the DNA ends for subsequent ligation in a process known as end processing [4, 21]. To minimize the risk of end processing mediated mutagenesis, increasing evidence suggests end processing is tightly regulated within a hierarchy to restrict processing factor activities to their respective incompatible ends and to allow less mutagenic factors preferential access to DNA ends [22–28]. This hierarchy is thought to be achieved via interactions between processing factors and the NHEJ core factors, as well as the DNA ends, with their improper regulation leading to detrimental effects on genome stability.

DNA polymerase λ (Pol λ) is a key factor involved in end processing during NHEJ and has been implicated in short patch base excision repair (BER) [29–31]. Recruited to the SRC through an interaction with Ku, Pol λ participates in gap filling of partially complementary DNA ends [32–34]. Importantly, Pol λ deregulation has been associated with cancer onset and progression with overexpression of Pol λ observed in 24% of human solid tumors [35]. Furthermore, a cancer-related variant of human Pol λ, R438W, exhibits reduced fidelity, leading to impaired DSB repair efficiency and an associated increase in chromosome instability [36]. Given the error-prone potential of Pol λ, restricting its activity is essential to prevent off-target nucleotide incorporation and safeguard genome stability.

**Figure 1.**
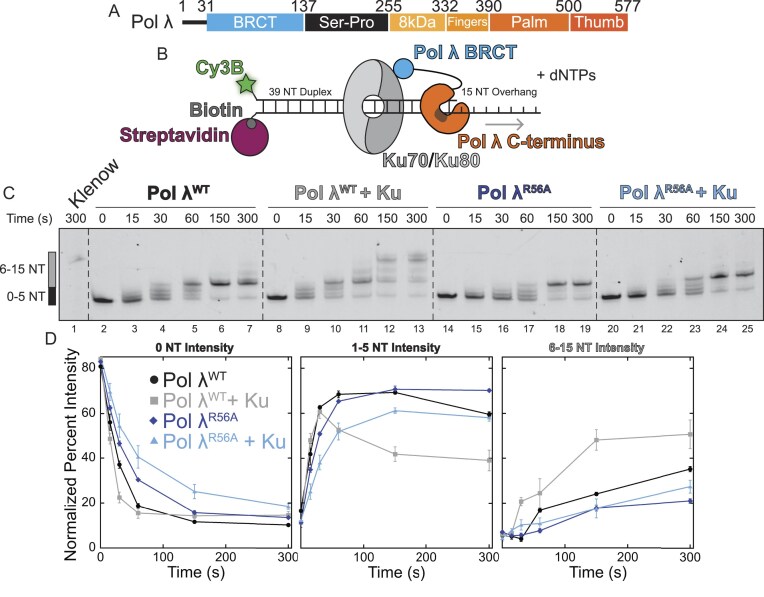
The presence of Ku increases Pol λ polymerase activity. **(A)** Primary structure of Pol λ showing the domain architecture. **(B)*** In vitro* Pol λ polymerase activity was assayed using a 5′-Cy3B labeled primer-template DNA junction blocked via a 3′-biotin-streptavidin to orient Ku loading. **(C)** Pol λ^WT^ incorporates 5 nucleotides over 5 min (lanes 2–7). The presence of Ku increases both the rate of nucleotide incorporation (compare lanes 4 and 10) and the length of Pol λ^WT^ extension (lanes 8–13). Ablating the interaction between Pol λ and Ku, using Pol λ^R56A^, shows no difference in nucleotide incorporation in the presence or absence of Ku (lanes 14–25). Full extension of the primer-template by Klenow is shown for comparison (lane 1). **(D)** Quantification of polymerase activity at different levels of nucleotide incorporation.

Compared to the other X family polymerases, Pol λ has unique features which specialize it for NHEJ. The N-terminal BRCT domain contains a KBM which recruits Pol λ to DSBs via interaction with Ku (Fig. 1A) [37]. This motif, consisting of α-helix1 of the BRCT domain, allows the polymerase to interact with Ku and XRCC4 [38, 39]. In particular, residue R56 (*Xenopus laevis*; R55 in *Homo sapiens*) is crucial for Ku interaction, with a mutation to alanine severely compromising the interaction [39]. The intrinsically disordered Ser-Pro rich region is unique to Pol λ and acts as a key regulatory region where phosphorylation modulates its chromatin association, polymerase activity and its ubiquitin-dependent degradation [40–43]. The remaining domains all contribute to the polymerase activity of Pol λ. The finger, palm, and thumb domains comprise the C-terminal catalytic domain and behave in a canonical manner to other DNA polymerases [44]. Pol λ preferentially incorporates complementary nucleotides [45] but is able to incorporate nucleotides adjacent to mismatches likely due to its inability to sense mismatched primer-template termini [46]. Interestingly, nucleotide incorporation is aided by the final noncatalytic domain, termed the 8kDa domain, which has a high affinity for the upstream 5′ phosphate and bridges the DNA gap. This upstream binding stimulates nucleotide incorporation by propelling the polymerase forward, in a process known as ‘scrunching’, though this activity is limited to gaps of shorter than 6 nucleotides, consistent with the gap sizes encountered during NHEJ [45, 47, 48].

Here, we investigate the role of Ku in regulating the activity of Pol λ during NHEJ. Using a minimal biochemical reconstitution of the *X. laevis* Pol λ-Ku complex, we ask how this interaction modulates polymerase activity. First, we demonstrate that Ku enhances Pol λ activity on a primer-template DNA junction. Next, we characterize a novel intramolecular interaction between Pol λ’s N-terminal BRCT domain and its C-terminal catalytic domain. Disrupting this interaction stimulates Pol λ polymerase activity, suggesting the polymerase is autoinhibited in its native state. Lastly, we find that the presence of Ku on a primer-template DNA increases the binding rate of Pol λ to DNA and increases Pol λ nucleotide incorporation, but does not affect its stability on DNA. Collectively, our results suggest a new mode of Pol λ regulation, where the BRCT domain acts in an autoinhibitory manner to restrict nonspecific polymerase activity outside of NHEJ, an inhibition which is relieved via Pol λ BRCT-Ku interaction within the SRC.

## Materials and methods

### Protein cloning, expression, purification, and labeling

The plasmid for Pol λ^WT^ was obtained from previous work [27]. Point mutants were generated using the QuikChange Lightning Kit (Agilent Technologies) following the manufacturers protocol from primers shown in Supplementary Table 1. Constructs needing domain removal or ybbR tag incorporation were generated by round-the-horn PCR using Q5 Polymerase (NEB) using the manufacturers protocol and primers shown in Supplementary Table 1. The plasmid for Ku70/80 was obtained from previous work and purified as previously described [17]. Reagents were purchased from Millipore Sigma unless otherwise noted.

Plasmids for Pol λ wild type and all constructs were transformed into BL21 (DE3) Rosetta pLysS cells by incubating 100 ng plasmid with 50 μl of cells on ice for 1 h before heat-shocking for 40 s at 42°C. After cooling on ice for 2 min, cells recovered in 100 μl SOC media while shaking at 37°C. After 1 h, cells were plated on LB-ampicillin plates and left to grow overnight at 37°C. Plates were removed the next morning and then a single colony was inoculated into a 10 ml Luria Broth (LB) (VWR) culture with 100 μg/μl Ampicillin and grown overnight shaking at 37°C. The next day, 2 × 1 l LB-Amp cultures were inoculated with 3 ml of overnight culture and allowed to grow at 37°C, shaking at 200 RPM, to an OD_600_ of 0.8 before inducing overnight with 1 mM Isopropyl β-D-1-thiogalactopyranoside (IPTG) at 16°C. Cells were harvested by centrifugation at 4667 × *g* for 5 min before resuspending in lysis buffer (20 mM HEPES, pH 7.5, 400 mM NaCl, 20 mM imidazole, 1 mM β-mercaptoethanol, 10% glycerol) and flash freezing in liquid nitrogen for storage at −80°C.

Resuspended cells were thawed and supplemented with a cOmplete, ethylenediaminetetraacetic acid (EDTA) free protease inhibitor cocktail tablet (Roche) pre-dissolved in 1 ml of deionized water. Cells were lysed on ice by sonicating for 2.5 min active, 1 s on, 3 s off, at 40% amplitude (FisherBrand Sonic Dismembrator) before separating the cellular debris via centrifugation at 47 850 × *g* for 1 h. The clarified supernatant was then incubated with a 2 ml bed volume of Ni-NTA (Qiagen) beads, pre-washed with lysis buffer, gently rotating at 4°C for 1 h. Resin was then pelleted by centrifugation at 1550 × *g* for two min to remove the supernatant before washing with 30 ml lysis buffer twice. Once washed, the resin was resuspended in 10 ml of lysis buffer to transfer to a 5 ml disposable column (Qiagen) and allowed to settle. Protein was eluted using 10 ml lysis buffer supplemented with 250 mM imidazole.

Unlabeled proteins were then cleaved by 5 μg/ml Ulp1 to remove the His-SUMO tag while dialyzing three times against 500 ml lysis buffer to remove excess imidazole overnight. The next day, proteins were passed through a 0.5 ml Ni-NTA column equilibrated in lysis buffer to remove the cleaved His-SUMO tag, Ulp1, and uncleaved protein. The flow through was then concentrated to 0.25 ml using 3 kDa Molecular Weight Cut Off Amicon Ultra concentrators (Millipore Sigma). Impurities were then removed from the concentrated protein by loading onto a Superdex 200 Increase 10/300 GL column (Cytiva) run on an ÄKTA Pure (Cytiva) in Size Exclusion Chromatography (SEC) buffer (20 mM HEPES, pH 7.5, 150 mM NaCl, 10% glycerol, 1 mM dithiothreitol). Pooled fractions containing pure protein were then concentrated for storage, if needed, before flash freezing in liquid nitrogen and storing at −80°C.

For labeling, proteins were concentrated down to 2.5 ml and buffer exchanged using a PD-10 column (Cytiva) into labeling buffer (25 mM Tris, pH 8, 150 mM NaCl, 20 mM imidazole, 10 mM MgCl_2_, 10% glycerol, 1 mM DTT) following the manufacturer’s protocol. After buffer exchange, protein was concentrated to at least 100 μM for labeling. A typical labeling reaction consisted of 1× protein, 1.5× CoA-conjugated dye, 5 μM Sfp synthase, and 15 μg/ml Ulp1. The reaction was allowed to rotate at 4°C overnight before passing through a 0.25 ml Ni-NTA column (Qiagen) to remove the cleaved His-SUMO tag, Ulp1, Sfp synthase, and uncleaved protein before concentrating the flow through to 0.25 ml using 3 kDa MWCO Amicon Ultra concentrators (Millipore Sigma). Concentrated labeling reactions were then loaded onto a Superdex 200 Increase 10/300 GL column (Cytiva) for SEC to remove impurities and excess dye, run using SEC buffer. Pooled fractions containing pure protein were then concentrated for storage, if needed, before flash freezing in liquid nitrogen and storing at −80°C. Reagents for ybbR labeling, CoA conjugates and Sfp synthase, were prepared as previously described [28, 49]. Purification gels for all proteins are shown in Supplemental Fig. 1D; nPol λ mutants were confirmed by mass spectrometry.

### DNA labeling and substrate preparation

All DNAs used in this study were purchased from Integrated DNA Technologies; DNA substrate sequences and uses are listed in Supplementary Table 2. Labeling of the amino-modified oligos (oBC148, oBC258) with Cy3B-NHS Ester (Cytiva Life Sciences) was performed overnight, rotating in the dark at room temperature in 100 mM sodium tetraborate, pH 8.5, 10% dimethyl sulfoxide (DMSO) at 1 mg/ml of oligo and 2 mg/ml Cy3B-NHS ester. After overnight incubation, the labeled DNAs were separated over a 20% acrylamide (19:1) 7M Urea-PAGE at 18 W for 1 h. The labeled DNA band was excised using UV shadowing before electroelution for 30 min using 3k MWCO dialysis tubing in 1× Tris-Borate-EDTA (TBE) buffer followed by ethanol precipitation and resuspension in 10 mM Tris–HCl, pH 8.

Primer-template DNAs were annealed by incubating a 1:1.05 ratio of Cy3B-labeled:biotinylated DNA strands at 150 nM in annealing buffer (10 mM Tris–HCl, pH 8, 50 mM NaCl, 1 mM EDTA) by heating to 95°C for 2 min and then decreasing the temperature by 1°C every minute until reaching 20°C. 3′ biotinylated DNAs (oBC172, oBC237, oBC259), were purchased from Integrated DNA Technologies (Supplementary Table 2). If needed, annealed samples had excess streptavidin added to a final concentration of 0.2 mg/ml to avoid loading multiple biotinylated substrates onto a single streptavidin. Annealed samples were run on a 12% acrylamide gel to confirm > 95% annealing (Supplementary Fig. S1A). It is of note that the substrate design using a 15 NT overhang is not ideal, as lengths longer than 4 nucleotide fill-in are rare. This template length was chosen to allow visualization of the polymerase activity while maintaining Ku loading onto the substrate.

### Bulk polymerase assays

Nucleotide incorporation by Pol λ was monitored by a shift in the fluorescently-labeled DNA upon running on a poly-acrylamide urea gel. Reactions consisted of 10 nM primer-template DNA, 12.5 nM Pol λ wild-type or mutant, 1 μM dNTPs or dTTP in reaction buffer [10 mM HEPES-KOH, pH 7.7, 50 mM KCl, 2.5 mM MgCl_2_, 1 mM DTT, 0.1 mg/ml bovine serum albumin (BSA), 250 mM sucrose]. Then, 5 μl of the reaction was taken at desired time points and quenched in 12.5 μl formamide stop buffer (98% formamide, 10 μM EDTA) before adding proteinase K (1.5 mg/ml final) for 1 h at 37°C. A 12% urea poly-acrylamide gel (7 M urea, run in 0.8× GTG buffer) was pre-run for 1 h at 18W before loading 7.5 μl of each sample (warmed to 95°C before loading) and running for 1 h. The gel was then imaged on a Typhoon 5 using laser settings for Cy3 dye before quantifying. Gels were quantified using the FujiFilm Multi Gauge software package by quantifying four sections from each lane: (i) a box for the unreacted substrate (0 NT Intensity), (ii) a box for incorporation of 1–5 nucleotides (determined by counting the number of discrete bands up from the unreacted substrate; 1–5 NT Intensity), (iii) a box for the incorporation of 6 or more nucleotides (determined in the same manner as 1–5 nucleotides incorporated; 6–15 NT Intensity), and (iv) a box containing the entire lane. The percent of each species per lane was calculated as the species of interest/entire lane. A minimum of a triplicate set of gels was quantified to plot the average number of nucleotides incorporated over time.

### Ku-DNA binding Electrophoretic Mobility Shift Assay (EMSA)

Binding of Ku to 12.5 nM primer-template DNA junction was assayed by titrating Ku70/Ku80 heterodimer up to 100 nM and incubating for ten min in reaction buffer. Samples were then diluted with glycerol for loading onto a 10% native acrylamide gel in 1× TBE and run for 60 min at 120 V. Gels were imaged on a Typhoon 5 for detecting the Ku/Cy3B-DNA complex using the Cy3 laser settings.

### PAE plot generation

PAE plots were generated from the .JSON files outputted by AlphaFold and visualized using the tool located at: https://thecodingbiologist.com/tools/pae.html

### Microscale thermophoresis (MST) assays

Microscale thermophoresis (MST) assays were performed in triplicate at the Harvard Medical School Center for Macromolecular Interactions (HMS CMI) on a Nanotemper Monolith NT.115Pico in Monolith Premium Capillaries at 50% excitation power, 60% MST power, 25°C with a target (FAM-ybbR-nPol λ) concentration of 50 nM and a ligand (Pol λ^Cat^) concentration ranging from 200 μM to 0.763 nM. Data were collected for a 1 s cold region start and a 20 s hot region. Data were fit over the time window from 1.5–2.5 s (S/N > 12) and analyzed using the MO.Affinity Analysis v2.3 software package.

### Single-molecule microscope and flow cell assembly

Single-molecule microscope set-up and flow cell assembly are the same as previously described [27, 28, 50]. Surface laser power density was 750 mW/cm^2^ for the 532 nm laser and 500 mW/cm^2^ for the 642 nm laser, as measured on the table using a Coherent FieldMate power meter.

### Single-molecule colocalization assay

Colocalization of Cy3B labeled DNA and Cy5 labeled Pol λ was monitored over time by first coating the flow cell with 0.2 mg/ml of streptavidin (Millipore Sigma). Excess streptavidin was then washed away using reaction buffer (10 mM HEPES-KOH, pH 7.7, 50 mM KCl, 2.5 mM MgCl_2_, 1 mM DTT, 0.1 mg/ml BSA, 250 mM sucrose) before adding 100 pM Cy3B-primer-template biotinylated DNA to coat the surface for 5 min. After washing out unbound DNA, 40 nM Ku was added, when needed, and allowed to bind DNA for 10 min. Flow cells were then primed with reaction buffer containing the oxygen scavenging system (5 mM protocatechuic acid, 0.1 μM protocatechuate-3,4-dioxygenase) and redox pair (1 mM ascorbic acid, 1 mM methyl viologen). Reactions monitoring binding of Pol λ to the DNA contained 10 nM Cy5-ybbR-Pol λ wild type or mutant in reaction buffer supplemented with an additional 0.5 mg/ml BSA (to prevent nonspecific binding), the oxygen scavenger and redox pair. After addition of Pol λ, images were taken continuously with an exposure time of 200 ms alternating excitation between 532 and 642 nm for 3 min per field-of-view (FOV) with three FOVs per flow cell imaged.

Colocalization data was analyzed using a freely available automated pipeline which aligns the cameras, corrects for drift, detects spots (based on circularity and distance to the nearest neighbor) and determines local background-corrected fluorescence intensities [51]. Colocalization events were called based on thresholds for Cy5 intensity and the distance between Cy3B and Cy5 foci, with thresholds set to minimize nonspecific binding at dark spots identified by the software (locations lacking DNA). Events were required to last for at least two frames with at least three frames between successive colocalization events. Background corrected fluorescence intensities were then analyzed in MATLAB (MathWorks) for changes in Cy5 intensity, corresponding to Pol λ binding events. These events were quantified by either the first binding event at the DNA foci, to determine the binding rate by fitting to a single exponential equation, or by their duration, to determine the dwell time of binding by fitting the histogram to an exponential decay.

### Single-molecule Förster Resonance Energy Transfer (FRET) nucleotide incorporation assay

The set-up to monitor FRET between Cy3B labeled DNA and Cy5 followed the same as the colocalization assay, but using DNA labeled −11 nucleotides away from the primer-template junction (Supplementary Table 2). Reactions monitoring nucleotide extension by Pol λ contained 10 nM Cy5-ybbR-Pol λ wild type or mutant and 1 μM dNTPs in reaction buffer supplemented with an additional 0.5 mg/ml BSA (to prevent nonspecific binding), the oxygen scavenger, and redox pair. After addition of Pol λ, images were taken at a continuous frame rate of 200 ms alternating excitation between 532 and 642 nm for 3 min per FOV with three FOVs per flow cell imaged.

FRET data was analyzed using the same user interface as the colocalization data [51]. Background corrected fluorescence intensities were then analyzed in MATLAB (MathWorks) to correct for bleed through from the Cy3B channel to the Cy5 channel, correct for Cy5 excitation from the Cy3 laser, correct for the gamma correction for Cy3B–Cy5 and eliminate photobleaching [28]. FRET positive events were counted in the analysis if they met the follow criteria: E_FRET_ was between 0.3 and 0.8 (linear R_0_ range), lasted at least 1 s (five frames), were shorter than 20 s (eliminate outliers) and had an overall negative slope throughout the observed FRET window. Events that passed these thresholds were fit in two ways: the histogram for the duration of the FRET events was fit to a single exponential decay to determine the dwell time and the magnitude of FRET change histogram was fit to a gaussian distribution to determine the average magnitude change.

## Results

### Pol λ interaction with Ku stimulates its polymerase activity

To test whether Ku stimulates Pol λ activity, we designed an *in vitro* bulk polymerase assay to compare Pol λ nucleotide incorporation on a primer-template substrate with or without the addition of Ku. We employed a 5′-Cy3B labeled DNA primer-template junction ensuring specific Ku loading onto the single-stranded DNA (ssDNA) side of the substrate (Fig. 1B and Supplementary Fig. S1A) by sterically blocking Ku loading to the dsDNA side through a 3′ biotin-streptavidin linkage [52–54]. When Ku was allowed to load onto both double- and single-stranded ends of the substrate, without the streptavidin block, we observed a modest, though reproducible, increase in Pol λ activity (Supplementary Fig. S1E). We ascribe this to Ku loading in an improper orientation and suggest the polarity of Ku relative to the DSB may be an important factor in allowing interacting partners access to the DSB during repair. The optimal concentration for efficient loading of a single Ku on the DNA substrate was determined via EMSA (Supplementary Fig. S1B).

Testing the activity of wild type Pol λ alone (Pol λ^WT^; purification shown in Supplementary Fig. S1D), we observed incorporation of ∼5 nucleotides over a 5-min incubation, as shown by the upward gel shift in Fig. 1C. The addition of Ku onto the DNA substrate increases polymerase activity and allows additional extension up to 15 nucleotides incorporated over 5 min. The quantification of the various nucleotide incorporation ranges are shown in Fig. 1D. As seen in Fig. 1C, a consistent accumulation of the 5-nucleotide extension product was observed across all conditions. To determine whether this accumulation was a sequence dependent consequence of our substrate design, we replaced our template region with a Poly-A ssDNA. This PolyA tail showed no accumulation of the 5-nucleotide extension intermediate while preserving the Ku-mediated polymerase activity increase (Supplementary Fig. S1F), suggesting the observed accumulation is substrate dependent.

To ensure that the Ku-mediated increase in polymerase activity is due to the direct interaction between Pol λ and Ku, and not just the presence of Ku on the substrate, we employed the previously characterized Pol λ^R56A^ mutant to ablate the interaction between Pol λ’s KBM and Ku (human Pol λ mutation R55A) [39]. As shown in Fig. 1C, the Pol λ^R56A^ mutant exhibits no increase in either the nucleotide incorporation rate or extension length in the presence of Ku, suggesting that the observed increase in activity of Pol λ^WT^ in the presence of Ku is due to the specific interaction between the Pol λ KBM and Ku.

### The Pol λ N-terminal BRCT domain binds into a pocket formed by the C-terminal catalytic domain

**Figure 2.**
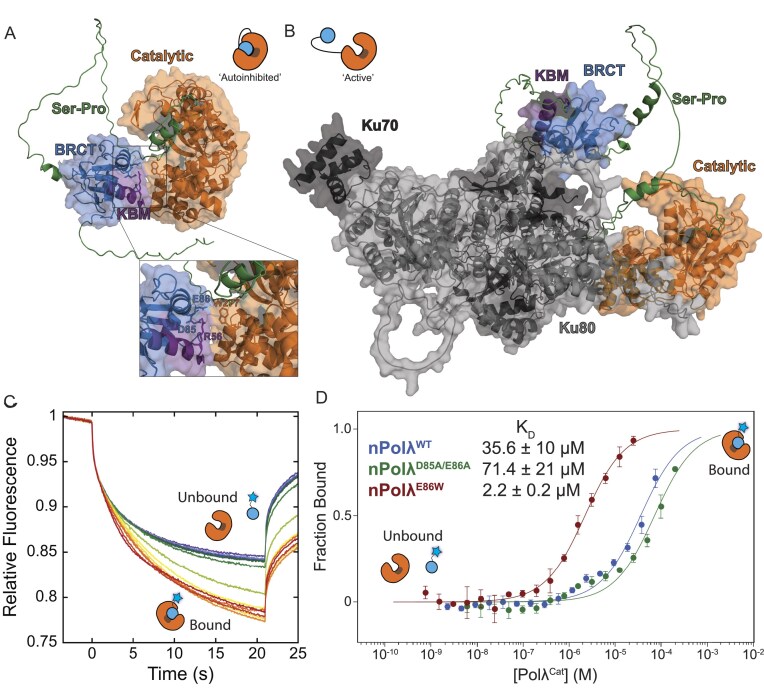
Characterizing the binding interaction between the N- and C-terminal domains of Pol λ. **(A)** AlphaFold model of full-length Pol λ showing a potential interaction between the N- and C-termini mediated by residues D85/E86 and W277/R278, respectively (inset). **(B)** AlphaFold model of full-length Pol λ and Ku 70/80, from https://predictomes.org, showing the known interaction between Pol λ KBM and Ku, which extracts the N-terminal BRCT domain from the catalytic domain of Pol λ. **(C)** Representative MST traces show a decrease in relative fluorescence upon binding of FAM-ybbR-nPol λ to Pol λ^Cat^. **(D)** MST dose-response curves of the interaction of nPol λ^WT^ (K_D_ = 35.6 ± 10 μM) or mutants with Pol λ^Cat^ show a decrease in affinity upon charge ablation (nPol λ^D85A/E86A^; K_D_ = 71.4 ± 21 μM) and an increase in affinity upon introducing a π-stacking interaction (nPol λ^E86W^; K_D_ = 2.2 ± 0.2 μM).

To better understand the mechanism by which Ku promotes an increase in Pol λ activity, we used AlphaFold 2 to generate a structural prediction of full-length *X. laevis* Pol λ in the presence and absence of Ku [55]. Alone, Pol λ is predicted to exist in a compact configuration with a potential intramolecular interaction between the N-terminal BRCT domain and the C-terminal catalytic domain that occludes the active site (Fig. 2A). This interface is mediated through residues Asp85/Glu86 and Trp277/Arg278 of the BRCT and catalytic domains, respectively (Figs. 2A and Supplementary Fig. S2A). Running an AlphaFold 2 model of Homo sapiens Pol λ shows a comparable interface mediated through residues Asp86/Glu87 and Trp274 of the BRCT and catalytic domains, respectively (Supplemental Fig. 2C and D).

Modelling full length *X. laevis* Pol λ in the presence of Ku, using AlphaFold-Multimer via predictomes.org, showed the interaction between Pol λ and Ku disrupts Pol λ’s N- to C-terminal intramolecular interaction, thereby opening the catalytic domain of the polymerase (Figs. 2B and Supplementary Fig. S2B) [56, 57]. Interestingly, the region of the BRCT domain interacting with both the catalytic domain and Ku are on adjacent helices, making simultaneous interaction with both binding partners sterically impossible. Overall, these structural predictions suggest Pol λ alone exists in an autoinhibited conformation, mediated by the intramolecular interaction between the N-terminal BRCT domain and the C-terminal catalytic domain. The presence of Ku opens the Pol λ catalytic pocket by sequestering the N-terminus, which may explain the observed enhancement of polymerase activity.

To validate this structural model, we generated mutants to either disrupt or stabilize the intramolecular interaction between the BRCT and catalytic domains of Pol λ. We did not attempt to mutate R278 given its known role in polymerization [58]. The neighboring W277A mutant severely attenuated polymerase activity (Supplementary Fig. S3A), likely due to its role in stabilizing the template strand during nucleotide addition [59]. To ablate electrostatic interactions between the domains, we cloned and purified a double mutant of D85 and E86 to alanine (D85A/E86A). In an effort to increase the affinity between the domains, we mutated E86 to tryptophan (E86W) to increase the stability of the intramolecular interaction via π-stacking with W277. To test the affinity of these binding partners, we separately purified the FAM-ybbR-labeled N-terminal BRCT domain (nPol λ, 1–144, Supplementary Fig. S1C) and the C-terminal catalytic domain (Pol λ^Cat^, 249–577, Supplementary Fig. S1C) and assayed their interaction using MST. Over a titration of Pol λ^Cat^, we observed a decrease in FAM-ybbR-nPol λ fluorescence due to binding between domains (Fig. 2C). Plotting the fluorescence change as the fraction bound, fits to a binding isotherm with a K_D_ of 35.6 ± 10 μM for the interaction between FAM-ybbR-nPol λ and cPol λ (Fig. 2D). Testing the mutant expected to disrupt binding between domains, nPol λ^D85A/E86A^, results in a K_D_ of 71.4 ± 21 μM, highlighting that the interaction between domains is mediated via the mutated residues. In contrast, testing the addition of a π-stacking interaction between subunits, nPol λ^E86W^, shows a 15-fold higher affinity of the intramolecular interaction with a K_D_ of 2.2 ± 0.2 μM. In addition, Pol λ^E86W^ polymerase activity was dramatically reduced compared to wild type and could not be rescued by the addition of Ku, suggesting the presence of Ku could not overcome the stabilized interface (Supplementary Fig. S3B). Given the necessity to remove the linker between the domains for this assay, these results may not represent the true affinity of the interaction between termini but do reflect the trend that the mutations would elicit in the full-length protein. Combined, these data validate the binding pocket predicted by AlphaFold using both gain and loss of function mutations at the interface.

### Disrupting the interaction between Pol λ N- and C-termini promotes polymerase activity

**Figure 3.**
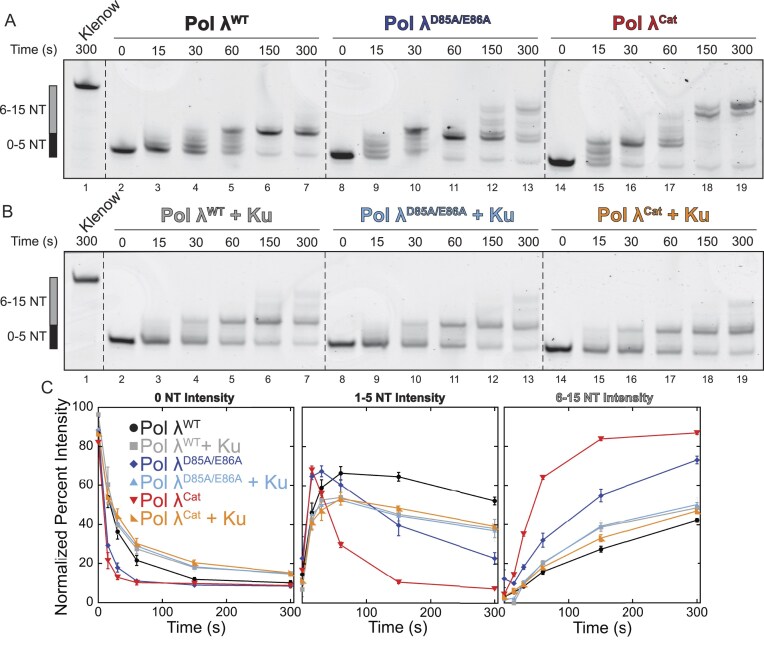
The intramolecular interaction between Pol λ N- and C-terminal domains autoinhibits nucleotide incorporation. **(A)** Comparison of the polymerase activities of Pol λ^WT^ (lanes 2–7), Pol λ^D85A/E86A^ (lanes 8–13), and Pol λ^Cat^ (lanes 14–19) shows a dramatic increase in both the speed and extension length for the mutants compared to wild-type. **(B)** In the presence of Ku, however, the wild-type and mutant polymerases show comparable activity. **(C)** Quantification of polymerase activity at different levels of nucleotide incorporation.

We sought to understand how the intramolecular Pol λ interactions mediate polymerase activity using our established polymerase assay by comparing the activity of Pol λ^WT^, the BRCT Pol λ^D85A/E86A^ double mutant, and an N-terminal deletion mutant, Pol λ^Cat^. Interestingly, both mutants showed a large stimulation in polymerase activity compared to wild type (Fig. 3A). This increase in activity supports our intramolecular interaction model by showing that reducing or completely removing the interaction between the N- and C-terminal domains activates polymerase activity and, therefore, acts as an autoinhibitory regulator in the absence of Ku. Importantly, both Pol λ^D85A/E86A^ and Pol λ^Cat^ showed an increase in activity comparable to wild type Pol λ in the presence of Ku with both an increased rate of polymerization and an increase in the length of extension (Fig. 3A and B). In the presence of Ku, however, both mutants showed slightly lower activity, possibly due to crowding on the DNA (Fig. 3B). The single point mutants, Pol λ^D85A^ and Pol λ^E86A^, had an intermediate phenotype between Pol λ with and without Ku (Supplementary Fig. S3C and D). Therefore, disruption of the intramolecular interaction between the N-terminal BRCT domain and the C-terminal catalytic domain as predicted in Fig. 2A significantly improves the polymerase activity in a similar way to the stimulation promoted by the addition of Ku to Pol λ^WT^. We propose that the interaction between the Pol λ N- and C-termini is an autoinhibitory mechanism to regulate Pol λ activity and specify its polymerase activity to NHEJ and, more specifically, to the SRC. Given the known interaction between Pol λ and Ku, we hypothesize that the observed increase in nucleotide incorporation in the presence of Ku is due to the release of Pol λ’s autoinhibition.

### Ku increases Pol λ binding to DNA and its stability

**Figure 4.**
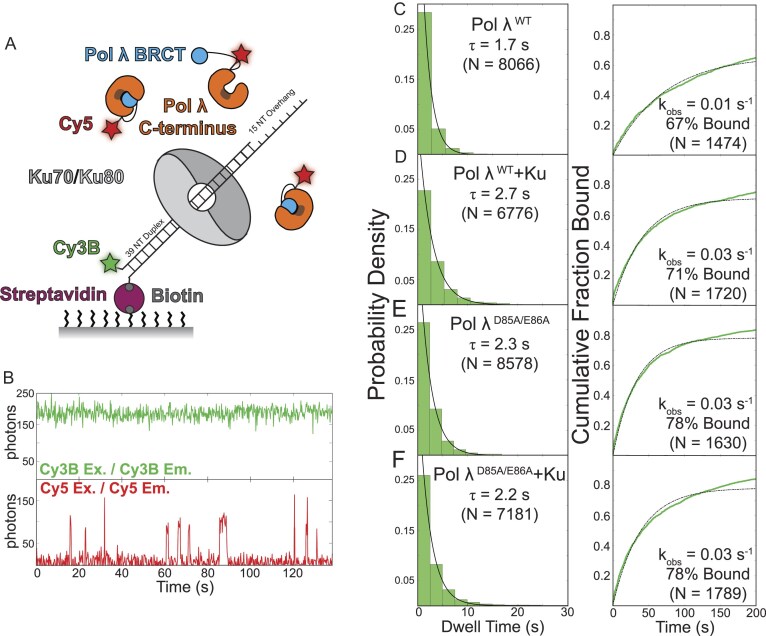
Ku increases the binding rate, but not the dwell time, of Pol λ on a primer-template DNA. **(A)** Schematic representation of the single-molecule colocalization assay using a 5′-Cy3B labeled primer-template DNA bound to the surface of the flow cell via a 3′-biotin-streptavidin linkage; Cy5-ybbR-Pol λ binding to the DNA was observed upon colocalization of the fluorescence signals. **(B)** Representative trace showing the number of photons emitted by the labeled primer-template DNA upon Cy3B excitation and emission (top panel), and the number of photons emitted by the labeled Pol λ protein upon Cy5 excitation and emission (bottom panel). Probability density (left) and cumulative fraction bound (right) plots of Pol λ^WT^**(C)** and Pol λ^D85A/E86A^**(E)** binding to the primer-template DNA in the absence and presence of Ku [panels (D) and (F), respectively].

We next sought to determine the mechanistic basis for the observed increase in Pol λ nucleotide incorporation in the presence of Ku. To this end, we designed a single-molecule colocalization assay to directly observe binding and dissociation of Pol λ to individual DNAs in the presence or absence of Ku. As shown in Fig. 4A, we attached the same Cy3B-labeled, biotinylated primer-template DNA substrate from the primer extension assay onto a streptavidin-coated coverslip. When needed, Ku was pre-incubated with the substrate to allow for loading onto DNA ends before flowing Cy5-ybbR-Pol λ into the flow cell. Labeled Cy5-ybbR-Pol λ proteins retain the same relative polymerase activity as the unlabeled versions (Supplementary Fig. S4A) and addition of BSA into the reactions prevented nonspecific binding to the flow cell (Supplementary Figs. S4B–D).

Single-molecule trajectories of Pol λ binding to the primer-template DNA were generated by observing colocalization between the Cy3B (DNA) and Cy5 (Pol λ) channels. Colocalization trajectories (an example shown in Fig. 4B) show multiple transient binding events of individual Pol λ molecules (lower panel, intensities above a threshold of 50 photons are considered colocalization events) to stably bound DNAs (upper panel). Quantifying the residence time of Pol λ on the DNA during colocalization events, we observe an increase in the dwell time of the polymerase in the presence of Ku (Pol λ + Ku; τ = 2.7 s) compared to its absence (Pol λ; τ = 1.7 s), suggesting that Ku plays a role in stabilizing Pol λ on DNA (Fig. 4C and D). Furthermore, Ku also increased the observed binding rate of Pol λ (Pol λ + Ku; *k_obs_* = 0.03 s^−1^; 71% bound) compared to binding in the absence of Ku (Pol λ; *k_obs_* = 0.01 s^−1^; 67% bound) (Fig. 4C and D). We theorized that the increased Pol λ DNA binding rate in the presence of Ku is mediated via the interaction of the BRCT domain which we tested using nPol λ, a minimal construct containing only the BRCT domain. In the absence of Ku, nPol λ was unable to bind DNA (Supplementary Fig. S4E); in the presence of Ku, nPol λ bound DNA at a comparable rate to Pol λ^WT^, although at a lower stability (nPol λ; *k_obs_* = 0.04 s^−1^; 52% bound; τ = 1.8 s) (Supplementary Fig. S4E). Combined, these data suggest that the initial recruitment of Pol λ to the primer-template junction is mediated through the BRCT-Ku interaction, which releases Pol λ autoinhibition by opening the catalytic domain. Next, positioned by its interaction with Ku, the Pol λ catalytic domain can rapidly bind the primer-template junction, which further stabilizes Pol λ on DNA.

According to this model, we expect that disruption of the intramolecular interaction between the N- and C-terminal domains of Pol λ would increase the binding rate and stability of Pol λ to the DNA in the absence of Ku, since the open catalytic pocket would be able to more readily engage the DNA junction. As anticipated, single-molecule imaging of the Pol λ^D85A/E86A^ mutant, with and without Ku, shows the same increase in binding rate as Pol λ in the presence of Ku (Fig. 4E and F) (Pol λ^D85A/E86A^; *k*_obs_ = 0.03 s^−1^; 78% bound) (Pol λ + Ku; *k*_obs_ = 0.03 s^−1^; 78% bound). Furthermore, the mutant shows an intermediate dwell time between that of Pol λ alone or in the presence of Ku, (Pol λ^D85A/E86A^; τ = 2.3 s; Pol λ^D85A/E86A^ + Ku; τ = 2.2 s), suggesting the role of Ku in extending Pol λ dwell time on the DNA is related to sequestering the N-terminus. Consistent with Pol λ^D85A/E86A^, we find that Pol λ^Cat^, which completely lacks the N-terminus, binds rapidly and stably in the absence of Ku (Pol λ^Cat^; *k*_obs_ = 0.04 s^−1^; 48% bound; τ = 2.6 s) while in the presence of Ku we see reduced binding, possibly due to steric clashing on the DNA (Supplementary Fig. S4F). Combined, the colocalization data support our hypothesis that the Pol λ N-terminal BRCT domain is autoinhibitory to the polymerase activity of Pol λ and that Ku mediates Pol λ-DNA binding via opening the catalytic domain and allowing it to engage the DNA primer-template junction.

### Ku increases the rate of nucleotide incorporation by Pol λ

**Figure 5.**
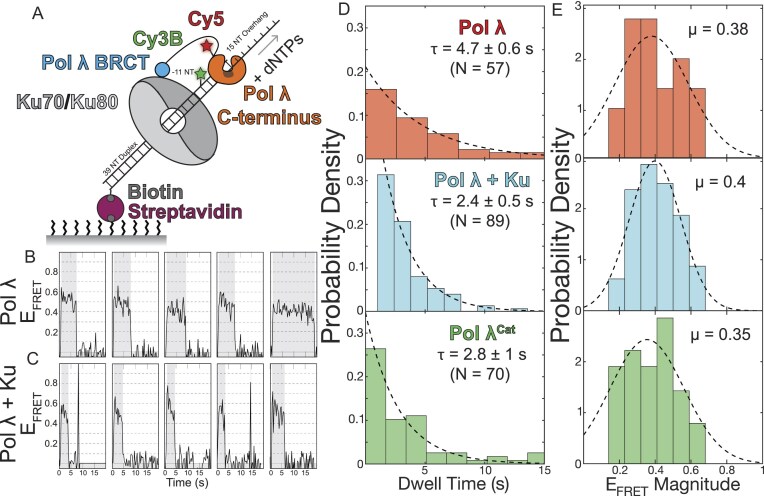
Pol λ polymerization rate is increased in the presence of Ku and the absence of its N-terminus. **(A)** Schematic representation of the single-molecule assay using a primer-template DNA duplex labeled at the -11 position from the junction bound to the surface of the flow cell via a 3′-biotin-streptavidin linkage; Cy5-ybbR-Pol λ binding to the DNA was observed as colocalization of the fluorescence signals. As shown by E_FRET_ over time, we observe a decrease in FRET between the DNA (donor) and Pol λ (acceptor) upon nucleotide incorporation both in the absence **(B)** or presence **(C)** of Ku. **(D)** The nucleotide incorporation time [gray areas in panels (B) and (C)] is the total time of E_FRET_ decrease and plotted as a probability density to determine the nucleotide incorporation dwell time. **(E)** The magnitude change of E_FRET_ (corresponding to 0.1 per nucleotide; Supplementary Fig. 5) is plotted as a probability distribution to determine the average number of nucleotides incorporated under each condition.

Our bulk assay shows Ku stimulates nucleotide incorporation, suggesting either Ku stimulates Pol λ processivity or that increased polymerase rebinding to the DNA allows faster nucleotide incorporation. To test these possibilities, we developed a single-molecule FRET assay to directly observe nucleotide incorporation on individual DNA substrates [60, 61]. As shown in Fig. 5A, the primer-template substrate was modified by moving the incorporated Cy3B dye from the 5′ of the primer strand to a nucleotide position -11 from the primer-template junction. This location allows for a starting FRET efficiency (E_FRET_) of ∼0.6 upon binding of Cy5-ybbR-Pol λ to the primer-template junction, which is within the linear range of the R_0_ for the Cy3B–Cy5 dye pair (Fig. 5B and C). Subsequent nucleotide incorporation should move the polymerase away from the donor dye and decrease E_FRET_. To determine how FRET efficiency changes upon nucleotide addition, we added subsets of dNTPs to generate well-defined products (note that the template sequence to pair the incoming nucleotides is ACGT, which allows specific incorporation of 1–3 nucleotides; Supplementary Fig. S5). Including just dTTP in the reaction resulted in a decrease in E_FRET_ of ∼0.1, including dTTP and dGTP showed a decrease in E_FRET_ of ∼0.2, while including dTTP, dGTP, and dCTP showed a decrease in E_FRET_ of ∼0.3. Collectively, these results indicate incorporation of individual nucleotides leads to a FRET efficiency decrease of 0.1 and strongly suggest that the reduction in E_FRET_ is due to nucleotide incorporation (Supplementary Fig. S5). Using this decrease in E_FRET_ as a metric for nucleotide incorporation, we asked how long Pol λ takes to add a set number of nucleotides to the primer DNA to understand how the rate of polymerization is affected by Ku.

Quantifying nucleotide incorporation by Pol λ in the single-molecule assay, we observed a decrease in E_FRET_ over time as represented within the shaded gray area (Fig. 5B). The length of the E_FRET_ decrease corresponds to the amount of time needed to incorporate nucleotides by Pol λ. Plotting these nucleotide incorporation dwell times as a probability shows that Pol λ spends 4.7 ± 0.6 s incorporating nucleotides when bound to the primer-template junction (Fig. 5D). In the presence of Ku, we see a decrease in the dwell time with Pol λ spending 2.4 ± 0.5 s incorporating nucleotides (Fig. 5C and D). Though Ku decreases the amount of time Pol λ is adding nucleotides, the change in E_FRET_ magnitude, which is representative of the number of nucleotides incorporated, is the same with Pol λ extending the primer by 3.8 or 4 nucleotides in the absence or presence of Ku, respectively (Pol λ, μ = 0.38/0.1 per nucleotide = 3.8 nucleotides; Pol λ + Ku, μ = 0.4/0.1 per nucleotide = 4 nucleotides; Fig. 5E). Therefore, with Ku bound, Pol λ adds the same number of nucleotides in less time, showing Ku stimulates nucleotide incorporation two-fold, but does not increase Pol λ processivity. To confirm that this increased incorporation rate was due to the release of Pol λ autoinhibition by Ku, we also tested Pol λ^Cat^ in the same assay and found comparable activity to Pol λ in the presence of Ku (Fig. 5D and E) (Pol λ^Cat^, τ = 2.8 s, μ = 0.35/0.1 per nucleotide = 3.5 nucleotides), confirming our hypothesis. Overall, our data suggest Ku stimulates Pol λ polymerase activity and increases Pol λ rebinding to the DNA, allowing for the increased nucleotide incorporation observed in our bulk assay.

## Discussion

DSB repair is essential to cell survival and genome maintenance [1]. The predominant DSB repair pathway, NHEJ, fixes these damages by directly ligating the strands back together. Frequently, however, DNA ends are damaged and thus must be acted on by DNA end processing factors to enable ligation [20]. Emerging evidence supports a hierarchical model of end processing in which high-fidelity enzymes are prioritized to mitigate additional error incorporation during NHEJ [25–27]. The regulation of error-prone end processing factors is critical for balancing the opposing goals of quickly repairing a wide array of damaged DNA ends and faithful preservation of the genetic code.

Pol λ is an error-prone end processing factor that is essential for efficient repair of gapped DNA substrates during NHEJ but can be mutagenic due to its role in nucleotide addition. Given this mutagenic potential, it is unsurprising that previous work has uncovered mechanisms by which Pol λ is regulated and targeted to the NHEJ synaptic complex. Post-translational modifications of the Ser-Pro region help modulate Pol λ’s activities. During the DNA damage response, ATM and DNA-PKcs phosphorylate Pol λ to localize it to chromatin and increase its polymerase activity once in proximity of broken DNA ends, respectively [40–43]. Importantly, Pol λ activity is further restricted to NHEJ by recruitment to DNA ends through an interaction of Ku with the Pol λ KBM, which is contained within the N-terminal BRCT domain. Our previous work showed that although Pol λ is recruited via its KBM to the LRC (prior to SRC formation), it is not active until SRC formation due to steric occlusion from the DNA ends by DNA-PKcs [27]. As the transition from the LRC to SRC requires engagement of the DNA ends by Lig4, all end processing factors are blocked by this initial Lig4 ligation attempt [28]. Structural studies of another NHEJ polymerase, Pol μ, observed it engaging DNA ends within the SRC after a conformational change of Lig4, which allows end processing factors access to DNA ends [33, 62]. On gapped DNA substrates, the 8 kDa domain of Pol λ reaches across the gap to interact with the upstream 5′ phosphate, which increases its polymerase activity [45, 47, 48]. In the context of the SRC, the 8kDa domain stimulates Pol λ-mediated repair of paired DNA ends where Pol λ can exploit any existing homology [27, 48].

**Figure 6.**
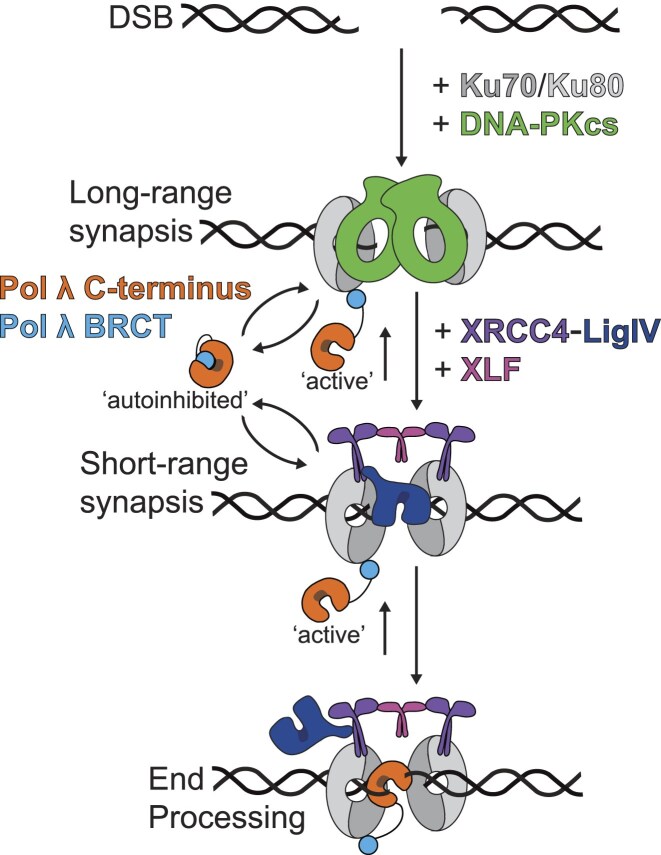
Model of Ku-mediated stimulation of Pol λ activity. In solution, Pol λ exists in an equilibrium between ‘active’ and ‘autoinhibited’ states, favoring the autoinhibited state. In the active state, Pol λ can independently bind DNA to begin polymerization. During NHEJ, rapid Ku binding to primer-template junction DNA ends creates a high affinity site for Pol λ recruitment. Interaction of the N-terminal BRCT domain of Pol λ with Ku shifts the Pol λ equilibrium to the active state. Combined, the active state of Pol λ and the high local concentration of primer-template DNA ends allows Pol λ to rapidly bind the junction and incorporate nucleotides.

Our data presented here provide a new regulatory mechanism to inhibit Pol λ outside of NHEJ via an autoinhibitory interface that is relieved by interaction with Ku (Fig. 6). We show that the polymerase exists in two conformations: the active and autoinhibited states, in which the autoinhibited state predominates in solution. In the active state, Pol λ can bind a DNA primer-template junction to incorporate nucleotides, though Pol λ is a slow and nonprocessive polymerase [45]. This polymerase activity is likely limited by the constant, transient interaction between the N- and C-terminal domains of Pol λ. Interaction with Ku not only recruits Pol λ to DNA ends but also extracts the N-terminus from the catalytic site to stimulate Pol λ nucleotide addition within the SRC. This increase in activity allows for site-specific nucleotide incorporation to further regulate the genome maintenance function of Pol λ. Once the N-terminus is engaged with Ku, the C-terminal catalytic domain of Pol λ is free to engage the local high concentration of primer-template. Sequestration of the N-terminus of Pol λ by Ku thus allows for faster binding of Pol λ to DNA and increases its rate of polymerization. Without this autoinhibitory mechanism, Pol λ may participate in low fidelity synthesis outside of its intended role in DNA end repair.

In addition to its role in NHEJ, Pol λ is also utilized in the short patch BER pathway, especially in conditions where Pol β is unable to complete repair, such as oxidatively damaged bases [29]. Interestingly, the BER scaffolding factor, XRCC1, stimulates Pol λ on gapped DNA substrates *in vitro* [63]. This behavior is similar to what we show here with the NHEJ core factor, Ku. We speculate that the interaction between XRCC1 and Pol λ relieves Pol λ autoinhibition in an analogous manner to Ku [63].

Overall, we propose a new mechanism of Pol λ regulation which tightly couples nucleotide incorporation to Ku binding and thus restricts polymerase activity to NHEJ in order to safeguard genetic integrity. It is notable that this additional level of activity regulation is essential for error-prone polymerases, but other processing factors that lack the ability to introduce genetic changes, such as PNKP or TDP1, likely do not require these additional regulatory mechanisms.

## Supplementary Material

gkag114_Supplemental_File

## Data Availability

Code for the initial single molecule imaging pipeline was previously published [51]. Raw single molecule traces are available upon request. Uncropped images, replicates of gel data, and downstream analysis code from this manuscript are available on Zenodo at https://doi.org/10.5281/zenodo.17379985

## Notes

Present address: Department of Cell Biology, Blavatnik Institute, Harvard Medical School, Boston, Massachusetts 02115, United States

Present address: Department of Immunobiology, Yale University School of Medicine, New Haven, Connecticut 06511, United States
