# Supplementary material for: DNA polymerase λ autoinhibition is relieved via Ku interaction during non-homologous end joining
Source: Nucleic Acids Res. 2026 Feb 17;54(4):gkag114. doi: 10.1093/nar/gkag114 (PMC12910108; doi:10.1093/nar/gkag114)
Supplement: gkag114_Supplemental_File [file gkag114_supplemental_file.pdf]

Supplemental Information to:

DNA POLYMERASE λ AUTOINHIBITION IS RELIEVED VIA KU INTERACTION DURING NON-HOMOLOGOUS END JOINING

Brandon C. Case[1], Leonardo Scoccia[2,*], Zhihan Zhao[3,#], Joseph J. Loparo[1,†]

[1] Department of Biological Chemistry and Molecular Pharmacology, Blavatnik Institute, Harvard Medical School, Boston, Massachusetts 02115, USA
[2] Department of Biology and Biotechnology, University of Pavia, Pavia, Italy; Institute of Molecular Genetics - National Research Centre (IGM-CNR), Pavia, Italy
[3] Department of Chemistry, Vanderbilt University, Nashville, Tennessee 37235, USA
[†]Correspondence should be addressed to joseph_loparo@hms.harvard.edu
*Present address: Department of Cell Biology, Blavatnik Institute, Harvard Medical School, Boston, Massachusetts 02115, USA
[#]Present address: Department of Immunobiology, Yale University School of Medicine, New Haven, Connecticut 06511 USA

Supplementary Figure 1:

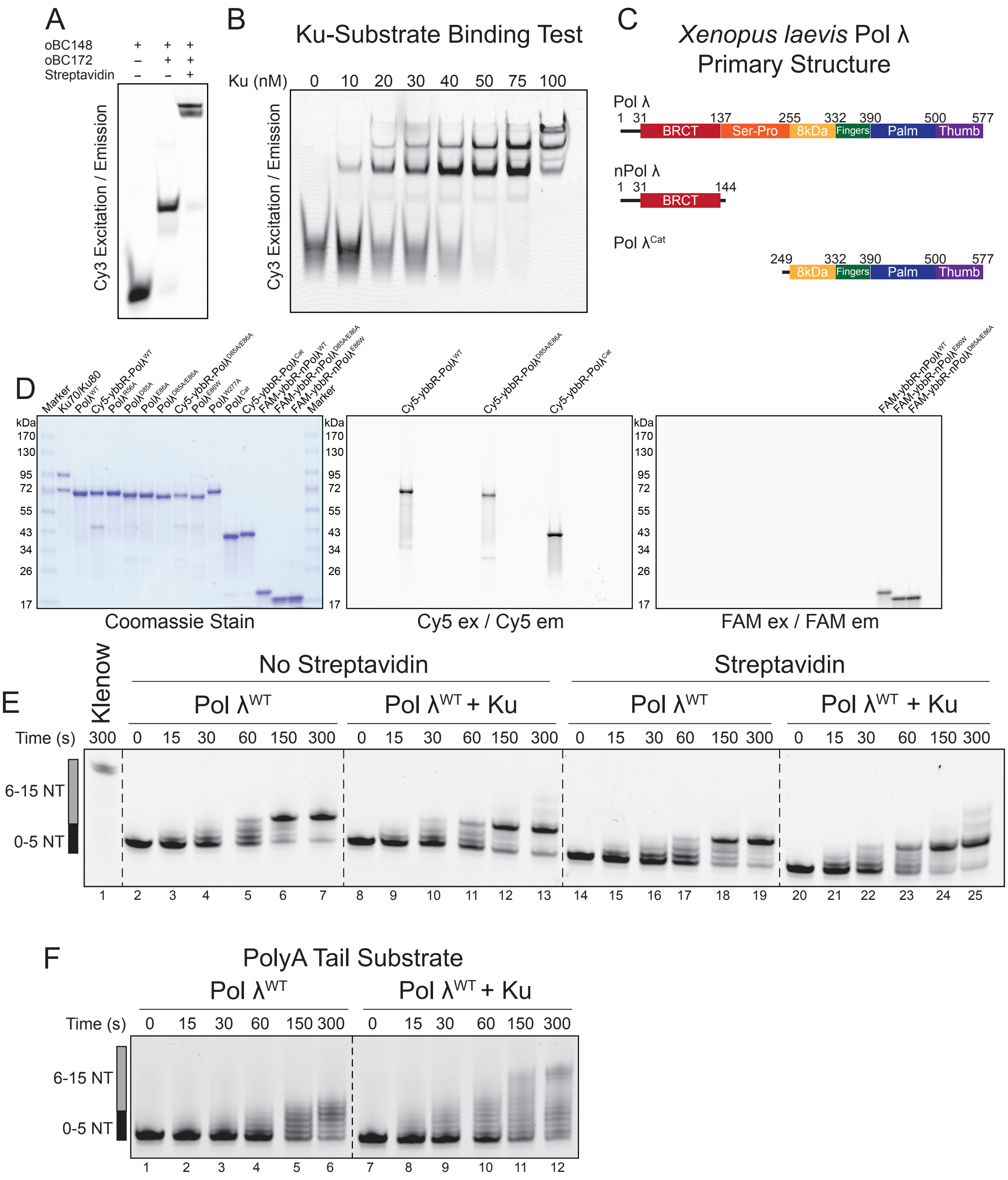

Supplementary Figure 1: Characterization of the *in vitro* Pol λ polymerase assay. (**A**) Annealing gel of the primer-template DNA substrate showing a shift of the Cy3B-oBC148 strand after annealing with biotin-oBC172, and again after incubation in the presence of streptavidin (sequences in Supplementary Table 2). (**B**) EMSA showing Ku binding to 12.5 nM primer-template DNA substrate over a range of Ku concentrations; 20 nM Ku was selected to maximize Ku binding and minimize overloading. (**C**) Primary structure of Pol λ constructs used in this study. Note ybbR-Pol λ

is labeled by inserting the ybbR sequence (DSLEFIASKLA) at position 238; both nPol λ and Pol λ^{Cat} ybbR constructs are labeled at their N-termini. (**D**) Acrylamide gel comparing the purity of the proteins used in this study and their degree of labeling. (**E**) Comparison of Ku stimulated Pol λ nucleotide incorporation in the presence or absence of streptavidin. (**F**) A 15 nucleotide Poly-A tail templated substrate, with an identical duplex region, shows a comparable Ku stimulated increase in activity to the heterogeneous tail substrate (sequences in Supplementary Table 2).

Supplementary Figure 2:

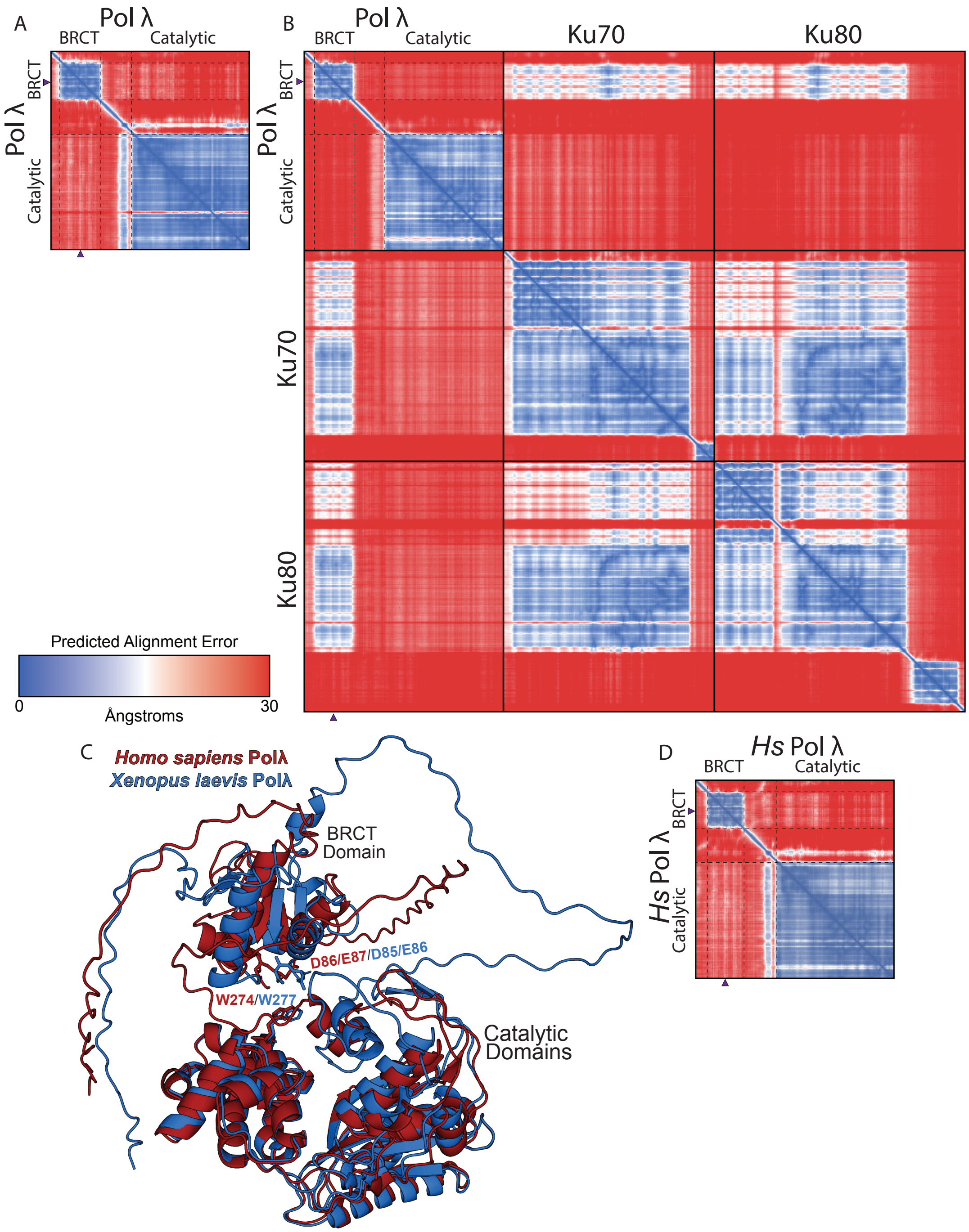

Supplementary Figure 2: Predicted alignment error (PAE) plots for Pol λ alone (**A**) or in the presence of Ku (**B**) highlighting the residues (D85 & E86, purple arrows) implicated in the intramolecular interaction between N- and C-termini. Plots were generated from the output .JSON files using an online tool at https://thecodingbiologist.com/tools/pae.html. (**C**) Overlay of AlphaFold 2 structures of full-length *Homo sapiens* and *Xenopus laevis* Pol λ showing comparable autoinhibited states. (**D**) PAE plot for *Homo sapiens* Pol λ.

Supplementary Figure 3:

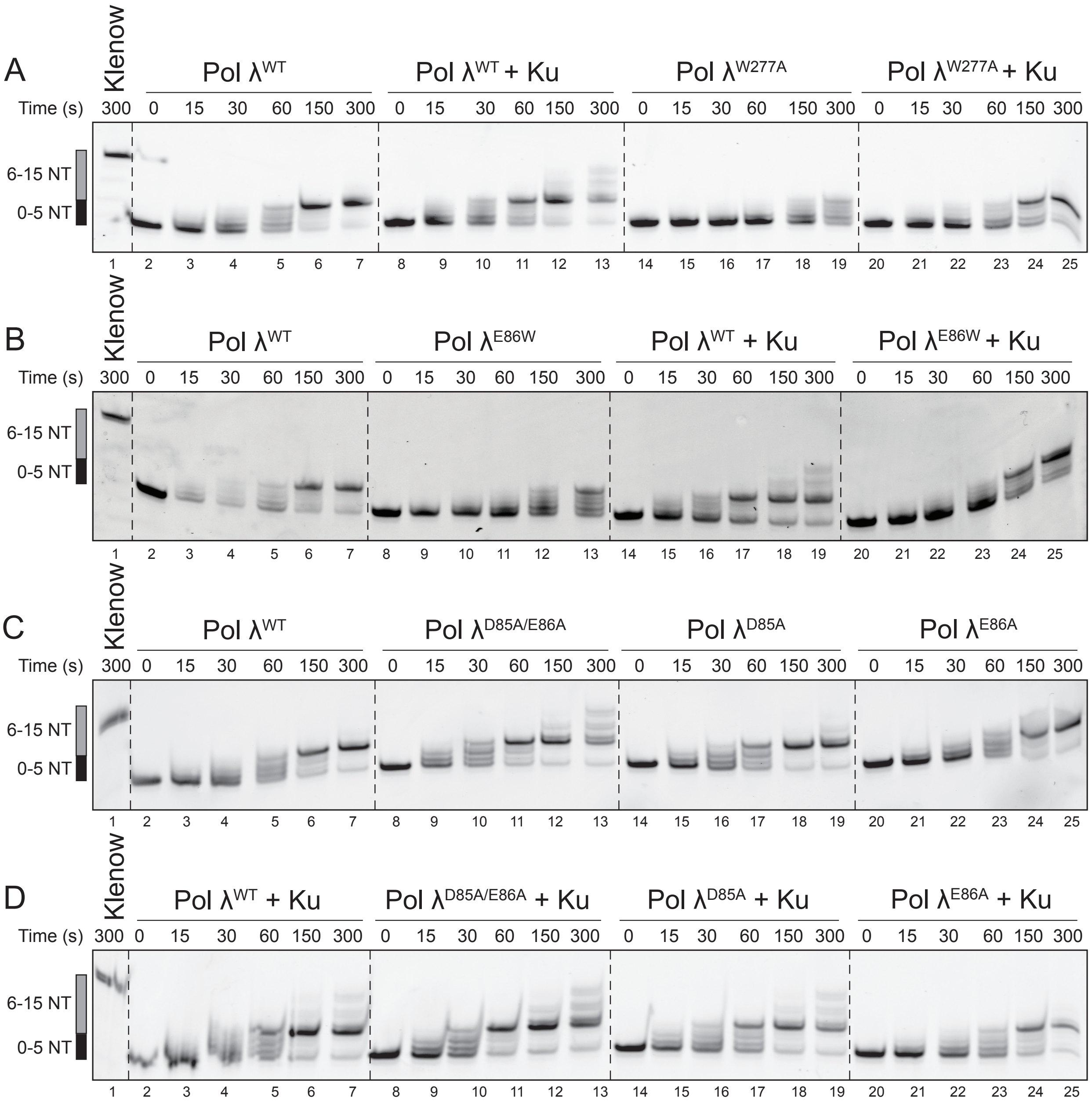

Supplementary Figure 3: Nucleotide incorporation of Pol λ binding interface mutants. (**A**) Pol λ^{W277A} polymerase activity is reduced compared to wild-type, likely due to the role of residue W277 in stabilizing the template strand. (**B**) Pol λ^{E86W} polymerase activity is reduced compared to wild-type, which we hypothesize is due to the stabilization of the binding interface between the N- and C-termini. (**C**) Both single mutants, Pol λ^{D85A} and Pol λ^{E86A}, show intermediate nucleotide incorporation between Pol λ^{WT} and Pol $\lambda^{D85A/E86A}$, suggesting that the double mutant shows an additive increase in polymerase activity. (**D**) In the presence of Ku, all constructs show an increase in activity, consistent with the role of Ku in biasing Pol λ to the open state.

Supplementary Figure 4:

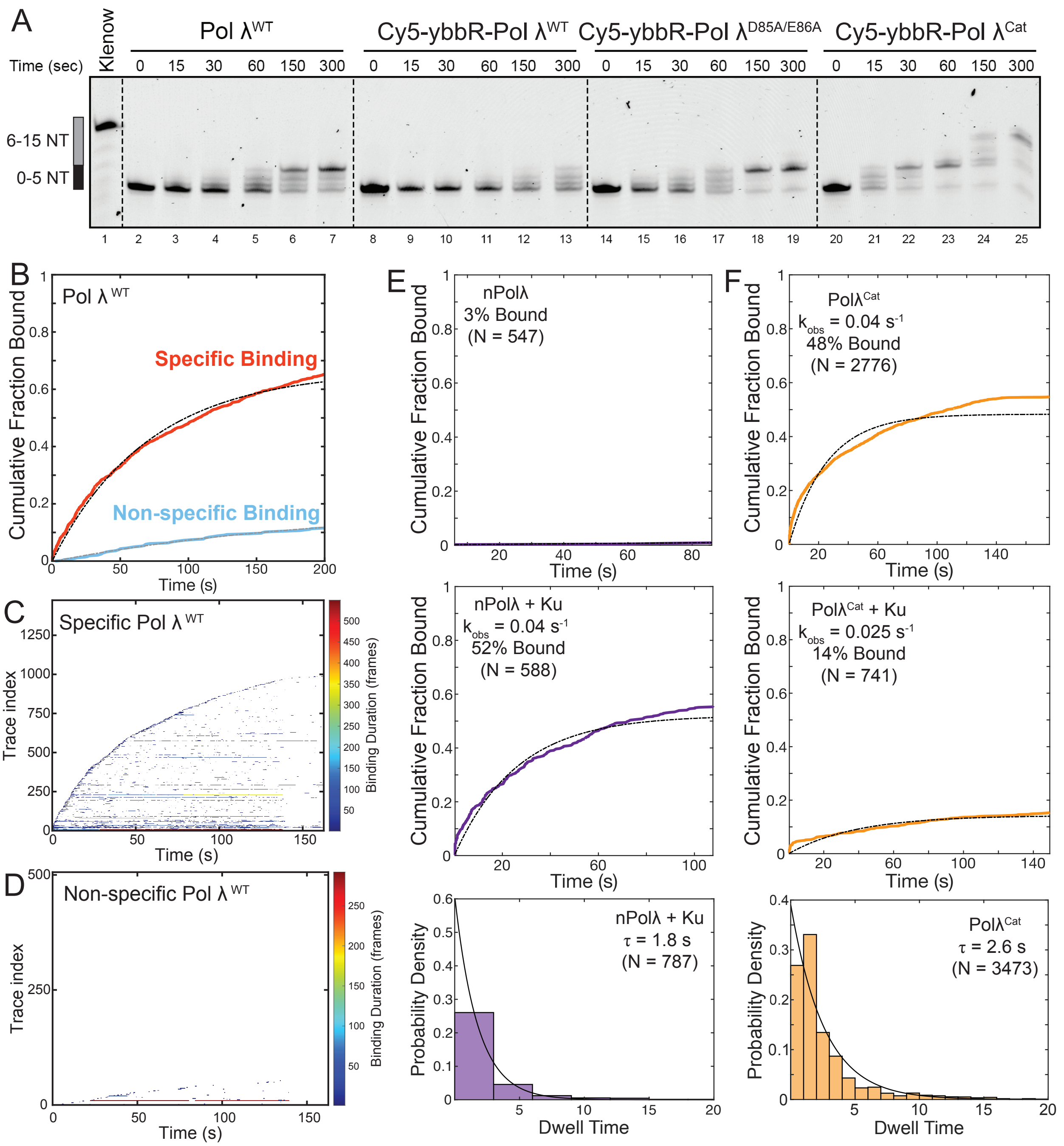

Supplementary Figure 4: Characterization of the single-molecule colocalization assay. (**A**) Pol λ^{WT} incorporates nucleotides faster than Cy5-ybbR- Pol λ^{WT} (compare lanes 2-7 with lanes 8-13). The labeled mutants, Cy5-ybbR- Pol $\lambda^{D85A/E86A}$ and Cy5-ybbR- Pol λ^{Cat}, however, incorporate nucleotides progressively faster than wild type, as seen with the unlabeled proteins (compare with Figure 3A). (**B**) Comparing specific (red) and non-specific (blue) binding of Cy5-ybbR-Pol λ^{WT} to DNA in the flow cell shows low levels of non-specific binding. Rastergram plots of the specific (**C**) and non-specific (**D**) binding to the flow cell show non-specific binding events are more transient and less frequent than the observed specific binding events. (**E**) Cy5-ybbR-nPol λ shows no binding to DNA in the absence of Ku and a comparable binding rate and dwell time to Pol λ^{WT} in the presence of Ku. (**F**) Cy5-ybbR-Pol λ^{Cat} binds to DNA with a comparable rate and dwell time to Pol $\lambda^{D85A/E86A}$ and displays little binding to DNA in the presence of Ku, possibly due to steric constraints.

Supplementary Figure 5:

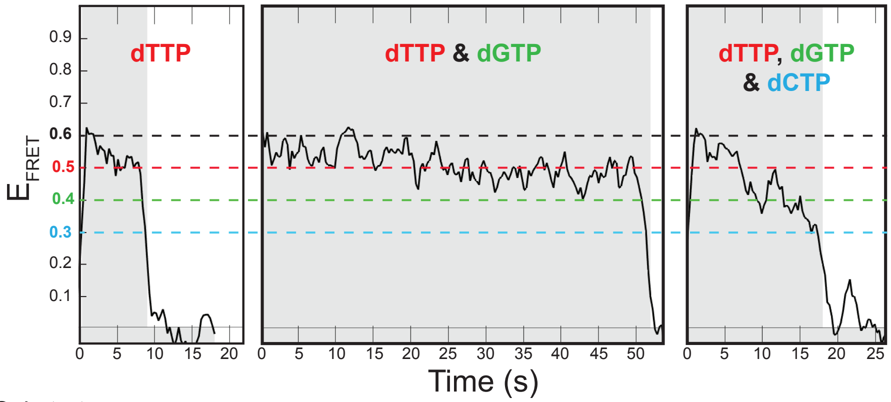

FRET Substrate:

TGG GCG CAA TCA CGA ATG AAT AAC CGT ATC GCC CTA TGC

ACC CGC GTT AGT GCT TAC TTA TTG GCA TAG CGG GAT ACG ACG TCC TGC TTC GGC

Supplementary Figure 5: Characterization of the single-molecule FRET nucleotide incorporation assay. Calibration of the E_{FRET} magnitude change as a function of nucleotide incorporation over time. FRET changes were measured under conditions that allowed incorporation of one (left), two (middle) or three (right) nucleotides on the FRET substrate by Cy5-ybbR-Pol λ. E_{FRET} magnitude change correlates to a decrease of ~0.1 per nucleotide added.

Supplementary Table 1: DNA plasmids and primers for this study

Plasmid	Protein Constructs	Oligo	Sequence	Source
pBC22	Polλ^{WT}	-	-	Stinson, 2021, Mol. Cell.
pBC36	Polλ^{Cat}	oBC76	5' - GAAAGCAAGAAGACGAATTACAACCAATTTATCA	This work
		oBC77	5' - TCCACCAATCTGTTCTCTGTGAGC	
pBC44	Polλ^{ybbR}	oBC73	5' - GCCAGTAAATTGGCTACTGGAAAGTGGGTCTGTGCTCACTCATC	This work
		oBC74	5' - GATGAACTCTAAGCTGTCGACATTATTTGTGACTTCATGGCTTTTCACAC	
pBC53	Polλ$^{Cat\ ybbR}$	oBC112	5' - GCCAGTAAATTGGCTGAAAGCAAGAAGACGAATTACAACCAATTTATCA	This work
		oBC113	5' - GATGAACTCTAAGCTGTCTCCACCAATCTGTTCTCTGTGAGC	
pBC66	Ku70/Ku80	-	-	Graham, 2016, Mol. Cell.
pBC75	Polλ^{R56A}	oBC164	5' - TGGCCTGGCCGCATCTGAAATT	This work
		oBC165	5' - AATTTCAGATGCGGCCAGGCCA	
pBC76	Polλ^{W277A}	oBC170	5' - AGGAGACCGCGCGAGAGCTTTGGG	This work
		oBC171	5' - CCCAAAGCTCTCGCGCGGTCTCCT	
pBC100	Polλ^{D85A}	oBC202	5' - GACGCACATTATAGTGGCTGAGCAAATGGACTGTG	This work
		oBC203	5' - CACGATCACAGTCCATTTGCTCAGCCACTATAATGT	
pBC101	Polλ^{E86A}	oBC204	5' - GACGCACATTATAGTGGATGCGCAAATGGACTGTGAT	This work
		oBC205	5' - CACGATCACAGTCCATTTGCGCATCCACTATAATG	
pBC102	Polλ$^{D85A/E86A}$	oBC200	5' - GACGCACATTATAGTGGCTGCGCAAATGGACTGTGATC	This work
		oBC201	5' - CACGATCACAGTCCATTTGCGCAGCCACTATAATGTG	
pBC105	Polλ$^{D85A/E86A\ ybbR}$	oBC73	5' - GCCAGTAAATTGGCTACTGGAAAGTGGGTCTGTGCTCACTCATC	This work
		oBC74	5' - GATGAACTCTAAGCTGTCGACATTATTTGTGACTTCATGGCTTTTCACAC	
pBC115	Polλ^{E86W}	oBC235	5' - CGCACATTATAGTGGATTGGCAAATGGACTGTGATCG	This work
		oBC236	5' - CGATCACAGTCCATTTGCCAATCCACTATAATGTGCG	
pBC117	nPolλ	oBC243	5' - TGACGAGCTCCTCGAGTAATAAGCTTGTCGAGCACC	This work
		oBC244	5' - TGCCCGGTCCTTCAAATCCAGGTACCTGTCTGG	
pBC127	nPolλ^{ybbR}	oBC87	5' - ATGAACTCTAAGCTGTCTCCACCAATCTGTTCTCTGTGAGC	This work
		oBC86	5' - CGCCAGTAAATTGGCTATGGAACCCAGAGGCATTGTTAAAG	
pBC128	nPolλ$^{D85A/E86A\ ybbR}$	oBC200	5' - GACGCACATTATAGTGGCTGCGCAAATGGACTGTGATC	This work
		oBC201	5' - CACGATCACAGTCCATTTGCGCAGCCACTATAATGTG	
pBC129	nPolλ$^{E86W\ ybbR}$	oBC235	5' - CGCACATTATAGTGGATTGGCAAATGGACTGTGATCG	This work
		oBC236	5' - CGATCACAGTCCATTTGCCAATCCACTATAATGTGCG	

Supplementary Table 2: DNA oligos for substrates in this study

Oligo	Strand	Sequence	Used in Figures:
oBC148	Primer	5'- /5AmMC6/TGGGCGCAATCACGAATGAATAACCGTACCGCCCTATGC	1, S1, 3, S3, 4, S4
oBC172	Template	5'- CGGCTTCGTCCTGCAGCATAGGGCGGTACGGTTATTCATTCGTGATTGCGCCCA/3Bio/	1, S1, 3, S3, 4, S4
oBC237	PolyA Template	5'- AAAAAAAAAAAAAAAAGCATAGGGCGGTACGGTTATTCATTCGTGATTGCGCCCA/3Bio/	S1F
oBC258	FRET Primer	5'- TGGGCGCAATCACGAATGAATAACCGTA/iAmMC6T/CGCCCTATGC	5, S5
oBC259	FRET Template	5'- CGGCTTCGTCCTGCAGCATAGGGCGATACGGTTATTCATTCGTGATTGCGCCCA/3Bio/	5, S5
